# Dr. Ira D. Hopkins

**Published:** 1914-04

**Authors:** 


Dr. Ira D. Hopkins, Buffalo, 1859, died at his home in Utica, February 23, aged 81. Death was due to a fall five days previously.

